# Maternal and Perinatal Outcomes of COVID-19-Positive Pregnant Women

**DOI:** 10.7759/cureus.26411

**Published:** 2022-06-28

**Authors:** Pragya Shree, Nupur Mittal, Soniya Vishwakarma, Vandana Verma, Virendra Pandey, Ekta Thadani

**Affiliations:** 1 Obstetrics and Gynaecology, Autonomous State Medical College Fatehpur, Mathura, IND; 2 Obstetrics and Gynaecology, Uttar Pradesh University of Medical Sciences, Saifai, IND; 3 Obstetrics and Gynecology, All India Institute of Medical Sciences, Raebareli, Raebareli, IND; 4 Obstetrics and Gynaecology, Kanti Devi Medical College and Research Centre, Mathura, IND

**Keywords:** nicu admission, fetal outcome, icu admission, maternal outcome, covid-19

## Abstract

Background

The risks of adverse maternal and perinatal outcomes are not very clear in coronavirus disease 2019 (COVID-19)-positive pregnant women. Therefore, this study aimed to determine the maternal and fetal outcomes in COVID-19-positive pregnancies.

Methodology

This prospective, cohort study was conducted in a tertiary care center over the period of one year. The study group comprised pregnant patients who presented with COVID-19 in the first and second waves of the pandemic. Maternal symptoms due to COVID-19 infection, comorbidities, number of admissions to the intensive care unit (ICU), and maternal mortality were noted for every patient. Perinatal outcomes were recorded in the form of intrauterine growth retardation (IUGR), mode of delivery, preterm deliveries, birth weight of newborns, neonatal intensive care unit (NICU) admissions, and neonatal mortality. Data analysis was done in the form of a variable percentage and mean ± standard deviation (SD).

Results

COVID-19-positive pregnant patients were mostly asymptomatic (48.07%). Term deliveries (37-40 weeks) were seen in 44 (89.8%) patients. The percentage of normal vaginal delivery was 74% and cesarean section was 24%. Out of 52 patients, two (3.8%) patients were admitted to the high dependency unit (HDU), one (1.9%) patient was admitted to the ICU, and 49 (94.3%) patients were in the isolation ward. Of the 49 live births, four (8.16%) newborns were admitted to the NICU. No neonatal death was recorded.

Conclusions

In this study, COVID-19-pregnant women were mostly asymptomatic. Neonates of COVID-19-infected women also mostly tested COVID-19 negative. More studies are needed with larger sample sizes to determine the effect of COVID-19 infection in pregnant women and neonates.

## Introduction

Coronavirus disease 2019 (COVID-19) is caused by severe acute respiratory syndrome coronavirus 2 (SARS-CoV-2) [1]. Cases of novel coronavirus-associated pneumonia were first reported in Wuhan City, China, in December 2019 [1]. The current pneumonia outbreak of COVID-19 was declared a pandemic by the World Health Organization (WHO) on March 11, 2020 [2]. The second wave came in mid-March of 2021, and the highest number of cases (144,829) was identified in India on April 9, 2021 [3]. Pregnant patients and newborns may be more susceptible to COVID-19 because of the physiologic changes that occur during pregnancy including cardiorespiratory and immune system changes, which may lead to altered response to SARS-CoV-2 infection during pregnancy [4]. As reported in many studies, the clinical course of COVID-19 pneumonia in pregnancy is similar to that in non-pregnant women, but the risk of adverse maternal and perinatal outcomes is not very clear in COVID-19-positive pregnant women. It has been hypothesized in some studies that upregulation of angiotensin-converting enzyme-2 (ACE-2) receptors in human gestation may actually be clinically protective but more studies are needed to confirm this hypothesis. Transmission of infection from the mother to the fetus like other viral diseases including human immunodeficiency virus (HIV), Zika virus, cytomegalovirus (CMV), etc., during antenatal, intrapartum, or postnatal at the time of breastfeeding can occur. Conflicting data exist regarding the vertical transmission of the virus [5-7]. Therefore, this study aimed to determine the maternal and fetal outcomes in COVID-19-positive pregnant women at our center.

## Materials and methods

This prospective, cohort study was conducted in the Department of Obstetrics and Gynaecology, Kanti Devi Medical College and Research Centre over a period of one year (May 2020 to May 2021). Ethical clearance was taken from the ethical committee of the institute (Kanti Devi Medical College and Research Centre, KDMCHRC/IEC/2020). The study group comprised pregnant patients who presented with COVID-19 in the first and second waves of the pandemic admitted to the ward. COVID-19-positive non-pregnant women, COVID-19-negative pregnant women, and women with known congenital fetal malformations were excluded from the study. After obtaining informed consent, cases were subjected to detailed history and physical examination. All necessary antenatal investigations and COVID-19-specific investigations, including complete blood count (CBC), C-reactive protein (CRP), liver function test (LFT), kidney function test (KFT), D-dimer, and chest X-ray posteroanterior (PA) view with abdominal shield were done on admission.

Maternal symptoms due to COVID-19 infection, comorbidities, number of admissions to the intensive care unit (ICU), and maternal mortality were noted for every patient. Perinatal outcomes were noted in the form of intrauterine growth retardation (IUGR), mode of delivery, preterm deliveries, birth weight of newborns, neonatal intensive care unit (NICU) admissions, and neonatal mortality.

**Figure 1:**
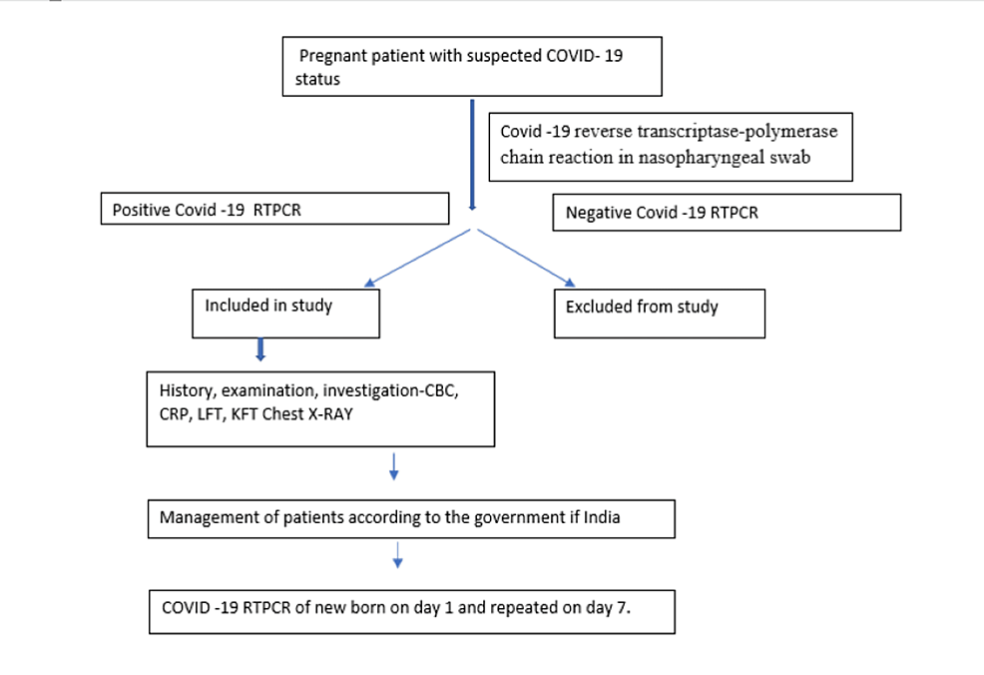
Flowchart of the methodology. COVID-19: coronavirus disease 2019; RTPCR: reverse transcriptase-polymerase chain reaction; CBC: complete blood count; CRP: C-reactive protein; LFT: liver function test; KFT: kidney function test

COVID-19 reverse transcriptase-polymerase chain reaction (RT-PCR) test was performed on swabs obtained from the nasopharynx for all newborns on the day of delivery and repeated on the seventh day of life. All newborns were allowed to breastfeed following preventive measures for COVID-19. Neonates were kept separately and given to mothers only during breastfeeding. Instructions were given to mothers regarding wearing a face mask to cover their nose and mouth during breastfeeding and hand hygiene (hand wash/sanitization) every time before breastfeeding. COVID-19-infected pregnant women were managed according to the Government of India guidelines. On the 10th day of admission, the SARS-CoV-2 RT-PCR test was performed on a swab from the nasopharynx of women again. Detection of COVID-19 was done by RT-PCR from a center authorized by the Government of India and state governments. We followed these pregnant women after discharge from the hospital till delivery and their maternal and fetal outcomes were noted. Figure 1 shows the flowchart of the study methodology.

All data were entered in a Microsoft Excel sheet and evaluated in the form of frequency, percentage, and mean ± standard deviation (SD).

## Results

In this study, 52 COVID-19, RTPCR-confirmed positive pregnant patients were observed, and maternal and neonatal outcomes were assessed. This number of patients was managed in our center during the first and second waves of the COVID-19 pandemic. Out of these, 49 patients were delivered, one patient was lost to follow-up, one patient had antenatal mortality, and in one patient spontaneous pregnancy loss was observed.

Patients’ ages ranged from 19 to 35 years, with the maximum number of patients (67.31%) in the age group of 19-25 years. The mean age of women in this study was 24.48 ± 3.23 years. Primigravida women were 40.38% while multigravida women were 59.62%. The majority (75%) of patients presented at 35-39 weeks of gestation (75.0%). Out of the 52 COVID-19-positive pregnant patients, only four (7.7%) had a history of recent out-of-state travel while three (5.8%) patients gave a history of contact with COVID-19 patients. In the rest of the 45 (86.5%) patients, the source of infection could not be identified and may be due to community transmission (Table 1).

**Table 1:** Demographic profile of study participants.

Parameters	Number of patients	Percentage (%)
Age (in years)		
19–25	35	67.31
26–30	13	25.0
31–35	4	7.69
Total	52	100
Parity		
P1	21	40.38
P2	21	40.38
>P3	10	19.23
Total	52	100
Gestational age on admission (weeks)		
<20	2	3.84
20–24	2	3.84
25–29	3	5.76
30–34	3	5.76
35–39	39	75.0
>40	3	5.76
Total (n)	52	100
History		
Travel history	4	7.7
Contact history	3	5.8
Unidentified	45	86.5
Total (n)	52	100

COVID-19-positive pregnant patients were mostly asymptomatic (48.07%). The most common presenting symptom was cough (44.23%), followed by fever (32.69%) and myalgia/malaise (19.23%). Less common symptoms were loss of taste and smell, nasal congestion, sore throat, dyspnoea, and headache in these patients (Table 2).

**Table 2:** Distribution of study participants according to symptoms.

Symptoms	Number of patients	Percentage (%)
Asymptomatic	25	48.076
Fever	17	32.69
Cough	23	44.23
Sore throat	5	9.61
Dyspnoea	4	7.69
Headache	2	3.84
Nasal congestion	8	15.38
Loss of smell/taste	8	15.38
Myalgia/malaise	10	19.23
Diarrhea	0	0

The majority (43) of COVID-19 mothers did not have any comorbidities, while four (7.69%) had hypothyroidism, two (3.84%) had anemia, and two women (3.84%) had gestational diabetes mellitus (GDM). Only one (1.92%) patient had pre-eclampsia (Table 3).

**Table 3:** Comorbidities in COVID-19-positive pregnant women. COVID-19: coronavirus disease 2019; GDM: gestational diabetes mellitus; DM: diabetes mellitus

Comorbidities	Number of patients	Percentage (%)
Pre-eclampsia	1	1.92
GDM/DM	2	3.84
Anemia	2	3.84
Hypothyroidism	4	7.69
None	43	82.69
Total patients	52	100

Term deliveries (37-40 weeks) were seen in 44 (89.8%) patients while only two (4.1%) patients had preterm delivery (34-37 weeks) and three (6.1%) patients were postdated. Of the 52 COVID-19-positive pregnant patients, 49 were delivered, one had a spontaneous pregnancy loss, and one patient was lost to follow-up. The percentage of normal vaginal delivery was 74% and cesarean section was 24%. The most common indication of cesarean section was pregnancies with previous cesarean section (58.33%), followed by fetal distress (16.66%). Only one patient expired during the antenatal period in this study (Table 4).

**Table 4:** Maternal outcomes of study participants.

	Number of patients	Percentage (%)
Time of delivery, gestational age (weeks)		
Preterm, <34	0	0
34–37	2	4.1
37–40	44	89.8
>40	3	6.1
Total (n)	49	100
Mode of delivery		
Abortion	1	2.0
Natural vaginal delivery	37	74.0
Lower segment cesarean section	12	24.0
Total	50	100
Indication of lower segment cesarean section		
Fetal distress	2	16.66
Previous cesarean section	7	58.33
Primigravida + breech	1	8.33
Severe intrauterine growth retardation	1	8.33
Second stage arrest	1	8.33
Total	12	100

Out of 52 patients, two (3.8%) patients were admitted to the HDU, one (1.9%) patient was admitted to the ICU, and 49 (94.3%) patients were in an isolation ward. Hospital stay was around 10-14 days in 44 (84.6%) COVID-19-positive women while eight (15.4%) women had a longer stay of more than 14 days (Table 5).

**Table 5:** Severity of COVID-19 and duration of hospital stay. COVID-19: coronavirus disease 2019

	Number of patients	Percentage (%)
Severity of COVID-19 symptoms		
Isolation ward	49	94.3
High dependency unit admission	2	3.8
Intensive care unit admission	1	1.9
Total (n)	52	100
Duration of hospital stay (days)		
10–14	44	84.6
>14	8	15.4
Total (n)	52	100

There was 100% live birth, and no fetal death was reported during the study. The maximum number of neonates (36.7%) had a birth weight of around 2.5-2.9 kg while six (12.3%) neonates had a birth weight of more than 3.5 kg and 10 (20.4%) newborns had a low birth weight of around 2.0-2.4 kg. Four (8.2%) newborns had low five-minute APGAR scores (4-6) while 45 (91.8%) newborns had an APGAR score of 7-10. Out of the total 49 live births only one (2.04%) newborn was COVID-19-positive and four (8.16%) newborns were admitted to the NICU (Table 6).

**Table 6:** Neonatal outcomes of study participants. COVID-19: coronavirus disease 2019

	Number of neonates	Percentage (%)
Outcome of delivery		
Intrauterine fetal demise	0	0
Live birth	49	100
Birth weight (kg)		
1.5–1.9	0	0
2.0–2.4	10	20.4
2.5–2.9	18	36.7
3.0–3.4	15	30.6
>3.5	6	12.3
APGAR Score (at five minutes)		
7–10	45	91.8
4–6	4	8.2
<3	0	0
Neonatal complications		
Neonatal intensive care unit admission	4	8.16
Neonatal death	0	0
COVID-19 positive	1	2.04
Total (n)	49	100

## Discussion

In this study, the maximum number of patients (67.31%) were in the age group of 19-25 years, whereas the mean maternal age was 30.8 years (range = 24-41 years) in a study by Yan et al. [7]. In this study, the majority of patients presented at 35-39 weeks of gestation, whereas in the study by Yan et al., the median gestational age on admission was 38 + 0 [7]. In this study, the percentage of primigravida was 40.38% while multigravida was 59.62%. Similar to this, in a study by Gupta et al., 18 (48.6%) women were primiparous and 19 (51.4%) were multiparous [8].

In this study, COVID-19-positive pregnant patients were mostly asymptomatic (48.07%). The most common presenting symptoms were cough (44.23%), fever (32.69%), and myalgia/malaise (19.23%). While in the study by Gupta et al., fever and myalgia were the most common presenting symptoms, followed by cough (10.8%) [8]. In this study, out of 52 women, 43 (82.69%) women did not have any comorbidities, while four (7.69%) patients had hypothyroidism, two (3.84%) patients had anemia, two (3.84%) had GDM, and only one (1.92%) patient had pre-eclampsia. While in the study by Islam et al., the most common complications noted were gestational hypertension, pre-eclampsia, and premature rupture of membranes. However, fewer women had anemia, GDM, or hypothyroidism [9].

In this study, term deliveries were seen in 44 (89.8%) patients, only two (4.1%) patients had a preterm delivery, and three (6.1%) patients were post-dated. The rate of preterm delivery was 13.5% in the study by Gupta et al. [8].

In this study, the percentage of normal vaginal delivery was 74.0% and cesarean section was 24.0% while 2.0% had an early first-trimester abortion. In the study by Singh et al., a cesarean section was performed among 78 (63.93%) women and 44 (36.07%) delivered vaginally [10]. However, high cesarean rates in COVID-19-infected pregnant women were also observed in some other studies [11,12].

In this study, the most common indication of cesarean section was previous cesarean section (58.33%), followed by fetal distress (16.66%), and the less common indications were primigravida with breech (8.33%), severe IUGR (8.33%), and second stage arrest (8.33%). A similar finding was reported by Singh et al. [10].

In this study, out of 52 patients, two (3.8%) patients were admitted to HDU while one (1.9%) patient was admitted to ICU, and 49 (94.3%) patients were in the isolation ward. One maternal mortality was found in our study. In the study by Singh et al., two patients were admitted to the ICU. Both were discharged in stable conditions. Maternal mortality was not found in the Singh et al. study [10].

In this study, the maximum number of neonates 18 (36.7%) had a birth weight of around 2.5-2.9 kg and 10 (20.4%) newborns had a low birth weight of <2.5 kg, while six (12.3%) neonates had a birth weight of more than 3.5 kg. In the Singh et al. study, low birth weight was observed in 13.5% of newborns [10]. In the study by Kalpana et al., most babies had a weight >2.5 kg [12].

In this study, only one (2.04%) newborn was COVID-19 positive out of the total 49 live births, and four (8.16%) newborns were admitted to a NICU. In the study by Singh et al., 40 (33.06%) babies were admitted to the NICU and two babies (1.65%) tested positive [10]. In the study by Gupta et al., all neonates were COVID 19 negative on day one of their life which ruled out the intrauterine transmission of COVID-19 from positive mothers during the third trimester [8]. The COVID-19 virus has been detected in vaginas, cervical, and anorectal swabs obtained from COVID-19-positive pregnant women in many studies, but we did not perform this test in our study [13,14].

There are some limitations of this study, such as its small sample size and single-center design. We did not have data related to COVID-19 testing in amniotic fluid, placenta, and cord blood samples. We did not have data on the long-term follow-up of neonates.

## Conclusions

In this study, COVID-19-positive pregnant women were mostly asymptomatic. Neonates of COVID-19-infected women also mostly tested COVID-19 negative whether the baby was born vaginally or by cesarean section. As the disease is new and long-term follow-up is required to study any residual or delayed effects on the newborns, it could not be ascertained whether COVID-19 causes any abnormality in the fetus. More studies are needed with larger sample sizes to find out the effect of COVID-19 infection in pregnancy and neonates. This knowledge can be helpful in antenatal counseling and deciding management protocols for safe and favorable maternal and neonatal outcomes.
